# AtROP1 negatively regulates potato resistance to *Phytophthora infestans* via NADPH oxidase-mediated accumulation of H_2_O_2_

**DOI:** 10.1186/s12870-014-0392-2

**Published:** 2014-12-30

**Authors:** Zhiwei Zhang, Fan Yang, Ren Na, Xiaoluo Zhang, Shuqing Yang, Jing Gao, Mingshou Fan, Yan Zhao, Jun Zhao

**Affiliations:** Department of Agronomy, Inner Mongolia Agricultural University, Huhhot, Inner Mongolia 010019 China; Institutes of Genetics and Developmental Biology, Chinese Academy of Science, Beijing, 100101 China

**Keywords:** AtRop1, Potato, Resistance, NADPH oxidase, H_2_O_2_ production, Jasmonic acid

## Abstract

**Background:**

Small GTPases are monomeric guanine nucleotide-binding proteins. In plants, ROPs regulate plant cell polarity, plant cell differentiation and development as well as biotic and abiotic stress signaling pathways.

**Results:**

We report the subcellular localization of the AtRop1 protein at the plasma membrane in tobacco epidermal cells using GFP fusions. Additionally, transient and stable expression of a dominant negative form (DN) of the *Arabidopsis* AtRop1 in potato led to H_2_O_2_ accumulation associated with the reduced development of *Phytophthora infestans Montagne* de Bary and smaller lesions on infected potato leaves. The expression of the *Strboh-D* gene, a NADPH oxidase homologue in potato, was analyzed by RT-PCR. Expression of this gene was maintained in DN-AtRop1 transgenic plants after infection with *P. infestans.* In transgenic potato lines, the transcript levels of salicylic acid (SA) and jasmonic acid (JA) marker genes (*Npr1 and Lox,* respectively) were analyzed. The *Lox* gene was induced dramatically whereas expression of *Npr1*, a gene up-regulated by SA, decreased slightly in DN-AtRop1 transgenic plants after infection with *P. infestans*.

**Conclusions:**

In conclusion, our results indicate that DN-AtROP1 affects potato resistance to *P. infestans.* This is associated with increased NADPH oxidase-mediated H_2_O_2_ production and JA signaling.

## Background

Potato (*Solanum tuberosum*) is the fourth largest crop in the world. Due to the high altitude, cold temperature and limited virus vectoring, Inner Mongolia has become the largest potato producing province in China. However, due to the absence of resistance genes to *P. infestans* in most cultivated potato varieties, potato late blight causes dramatic yield losses in Inner Mongolia [1,2]. Therefore, one of the major challenges for potato breeders is to decipher the resistance mechanisms to *P. infestans* and generate resistant cultivars through the combination of traditional and molecular breeding approaches. Numerous studies have investigated the molecular basis of quantitative resistance to pathogens [3], the identification of dominant resistance genes in potato [4,5], the pathogen invasion mechanisms [6,7], as well as potato resistant signal molecules [6,8]. Previous studies also indicated that salicylic acid (SA), jasmonic acid (JA) and defense genes such as *PR, StPK1*, and *StLRPK1* are involved in resistance to potato late blight [9-11]. However, an understanding of how small G proteins regulate resistance to *P. infestans* in potato is lacking.

Small GTPases are monomeric guanine nucleotide binding proteins [12]. Rho GTPase, one branch of the small GTPase Ras superfamily, contains three related subfamilies: Rho, Rac, and Cdc42 [13,14]. In yeast and mammalian cells, Rho GTPases have multiple roles in plants, regulating the cytoskeleton reorganization, cell polarity, cell wall synthesis, hydrogen peroxide (H_2_O_2_) production, cell cycle and differentiation [15-18]. Plants have evolved a distinct class of small GTPases named Rho-related GTPase (ROPs), which are very similar to Racs (a subfamily of Rho GTPase) from mammalian cells [19-21]. Plant ROPs not only exhibit high sequence similarity with mammalian Rho GTPases, but also possess similar functions [20,22,23]. Like their mammalian counterparts, ROPs are activated through guanine nucleotide exchange factors (GEFs) by exchanging GDP for GTP, whereas they are inactivated by GTPase-activating proteins (GAPs) and stimulate GTP hydrolysis to GDP. Guanine nucleotide dissociation inhibitors (GDIs) keep ROPs in an inactive form by inhibiting the release of GDP [19-21]. ROPs cycle between the GTP-binding form and the GDP-binding form, thus regulating a variety of cellular responses [24]. To date, several plant ROP genes have been identified, including the 11 Arabidopsis ROP genes [19,25,26], 7 rice genes and 9 maize genes [27]. The proteins encoded by these ROP genes regulate multiple signaling pathways, leading to a diverse array of cellular responses such as cell polarity/tip growth, cytoskeleton reorganization, secondary wall formation and plant defense [20,22,23,28].

Rho-related GTPases are clearly involved in the establishment of plant defense. In rice, OsRac1 positively regulates the defense response to *Magneporthe grisea via* H_2_O_2_ accumulation, achieved through the regulation of NADPH oxidase activity [29-32]. OsRacB, OsRac4 and OsRac5 act as negative regulators in the establishment of resistance to rice blast [33-36], but OsRac6 regulated rice resistance in a positive manner [36]. In mammalian cells, overexpression of the dominant positive conformation of ZmRac (cloned from maize) also results in an increase in the production of superoxide and other ROS molecules [37]. Overexpressing the GhRac13 gene (from cotton) in Arabidopsis and HsRac1 (from humans) in soybean inhibits H_2_O_2_ production [38,39]. In Arabidopsis, AtROP2 and AtROP11 transgenic plants exhibit increased resistance to the *Pseudomonas syringae* pv. *Tomato* (Pst) *DC3000 ( P. syringae)*. However, AtROP10 has the opposite effect on resistance to bacteria [40]. In barley, silencing HvRacB increases resistance to powdery mildew by reducing fungal haustorium establishment in a cell-autonomous and genotype-specific manner [41]. However, stable overexpression of CA-HvRACB, CA-HvRAC1, and CA-HvRAC3 (active conformation) in barley led to enhanced susceptibility to powdery mildew [33,42,43]. In tobacco, overexpression of the *MsRac1* gene results in cell death, thus leading to the development of brown necrotic lesions [44]. In addition, using the RNA interference silencing approach in *Medicago truncatula* plants indicates that MtROP9 plays a key role in ROS-mediated early infection signaling [45]. All of the above results demonstrate that ROPs play an important role (positively and negatively) on the establishment of plant defense.

Reactive oxygen species(ROS) including superoxide (O^2−^), hydrogen peroxide (H_2_O_2_), hydroxyl radical (HO·) and singlet oxygen (^1^O_2_), which generated by plasma membrane NADPH oxidases play pivotal roles in the defense response, and are thought to act as secondary messengers for the induction of resistance responses, such as the increased expression of defense genes and the induction of hypersensitive cell death known as the hypersensitive response (HR) [46,47]. Rapid production of ROS is one of the early events during incompatible interactions between plants and pathogens [46,48]. In *Nicotiana benthamiana* inhibiting ROS accumulation led to reduced resistance to *P. infestans* [47]. ROS in soybean cells may interact with nitric oxide to trigger the HR, thus effectively restricting pathogen growth [49]. In Arabidopsis, induction of ROS lead to the hypersensitive cell death response and enhanced its resistance to Pst and *Hyaloperonospora arabidopsidis* [50]. Increasing ROS production in rice induces HR-like responses, greatly contributing to reduction in the size of disease lesions caused by a virulent race of the rice blast fungus and altering expression of defense-related genes [51].

Many studies have shown that SA (salicylic acid) and JA (Jasmonic acid) are key signaling molecules which regulate plant resistance by inducing the expression of a series of defense genes,such as *PR-1*, *PAD4*, *EDS1,* and *PDF1.2* [52]. SA plays a role in the establishment of resistance to biotrophic pathogen infection and systemic acquired resistance (SAR) [53]. The non-expressor of PR gene 1 (*NPR1*) is up-regulated by SA accumulation during pathogen infection and is routinely used as a marker gene to track SA-mediated signaling pathways [54]. A recent study established that SA is required for basal defense such as pathogen triggered immunity (PTI) in potato against *P. infestans*; SA reduction renders plants more susceptible to *P. infestans* possibly due to lower *PR* gene expression [55]. Increasing SA levels in potato led to higher resistance to *P. infestans* [56]. JA is also believed to play an important role in the establishment of plant resistance to pathogens and in response to various stresses [57,58]. In plants, JA acts as a key signaling component involved in the establishment of induced systemic resistance (ISR) [59]. Lipoxygenase (Lox) is a key enzyme in the JA synthesis pathway, and the expression of the *Lox* gene is highly correlated with JA accumulation in response to various stresses [6]. *Arabidopsis thaliana* mutants impaired in the JA signaling pathway are always compromised in their resistance to necrotrophic pathogens [60]. JA acumulation was also observed after Pst DC3000 infection [59]. Silencing the allene oxide cyclase (*AOC*) or OPDA reductase 3 (*OPR3*) genes, both coding for enzymes involved in JA biosynthesis, also perturbs the potato response to Pep-13, a pathogen-associated molecular pattern (PAMP) from *P. infestans*, by reducing the accumulation of ROS and hypersensitive cell death. SA accumulation has been observed in JA deficient plants [6], indicating the existence of a cross talk between the JA and SA signaling pathways in the establishment of potato resistance.

We analysed AtRop1, a small G protein in *Arabidopsis* and its role in the establishment of resistance to potato late blight. Our results demonstrate that transient or stable overexpression of an inactive form of AtRop1 (DN-AtRop1) enhances potato resistance to *P. infestans* infection, a resistance associated with the accumulation of H_2_O_2_ mediated by a NADPH oxidase homologue gene. This suggests that H_2_O_2_ plays a crucial role in AtRop1-mediated potato resistance to *P. infestans* infection. Additionally, preliminary results indicate the involvement of JA in AtRop1-mediated potato resistance to late blight.

## Results

### Subcellular localization of AtRop1

**Figure 1:**
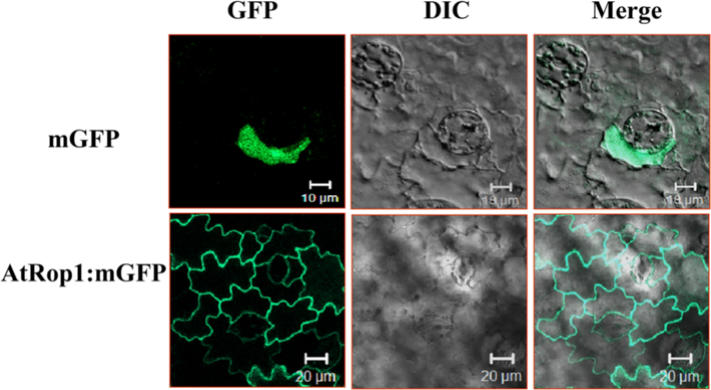
**The subcellular localization of AtRop1.** Transient expression of mGFP: AtRop1 in tobacco epidermal cells; differential interference contrast (DIC), refers to bright-field images of the cells.

To determine the subcellular localization of AtRop1, a mGFP:AtRop1 construct was transiently expressed in tobacco epidermal cells and the GFP signal detected under fluorescence microscopy. As shown in Figure 1, the green signal was observed only on the plasma membranes (PM), while the GFP alone (as control) was visualized mainly in the cytoplasm. Hence, AtRop1 is localized at the PM.

### Transient expression of a dominant inactive conformation of AtRop1 (DN-AtRop1) enhance potato resistance to *P. infestans*

**Figure 2:**
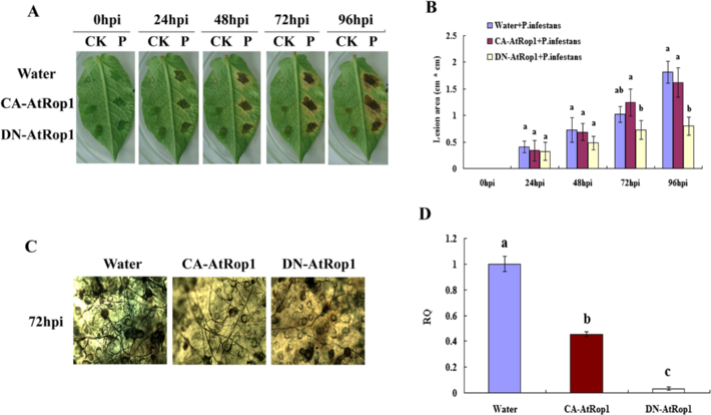
**Transient expression of At-Rop1 negatively regulated potato resistance to** ***P. infestans*** **.** *Agrobacterium tumefaciens* (LB4404) carrying CA-AtRop1 and DN-AtRop1constructs were infiltrated into potato leaves at a concentration of OD_600_ = 0.5. **A**. Development of lesion size on potato leaves after inoculation with *P. infestans* (5 × 10^5^ zoospore/mL). The left part of the leaf was used as a control by inoculating with water; the right part was inoculated with *P. infestans*. **B**. After transient expression of CA-AtRop1 and DN-AtRop1, the lesion area (length by width) was measured at different time point after inoculation with *P. infestans*. Each value represents the average of three lesions (cm^2^ ± standard deviation). Levels not labeled with the same letter are significantly different at the *P < 0.05* level based on the ANOVA. **C**. The development of *P. infestans* mycelium at sites where CA-AtRop1 and DN-AtRop1 were transiently expressed was quantified by trypan blue staining. **D**. The development of *P. infestans* at transient expression sites 72hpi was quantified by real-time PCR by comparing levels of the specific repetitive DNA sequence of *P. infestans* (as measure for fungal biomass) relative to potato *EF-1α* gene (for equilibration). Levels not labeled with the same letter are significantly different at the *P < 0.05* level based on the ANOVA. Bars represent averages with standard deviation of three technical replicates.

To determine the importance of AtRop1 in the potato response to *P. infestans*, *Agrobacterium tumefaciens* (LB4404) strains carrying either CA-AtRop1 (constitutively active) or DN-AtRop1 (dominant negative) were infiltrated into potato leaves. Twenty-four hours later, *P. infestans* (5 × 10^5^ zoospore/mL) was inoculated at the infiltration sites and the lesion sizes were measured at different time points after inoculation. As shown in Figure 2A, lesions were observed on potato leaves 24 hours post-inoculation (hpi) and the size expanded gradually. Differences in lesion size were observed at 72 hpi and 96 hpi. The lesions at the infiltration sites transiently expressing DN-AtRop1 were much smaller than lesions at the sites expressing CA-AtRop1 (Figure 2A). Quantification lesion size showed that the rate of lesion expansion after DN-AtRop1 infiltration was much slower compared with that at CA-AtRop1 infiltration sites (Figure 2B)*.* These results suggest that AtRop1 may be involved in potato resistance to *P. infestans*.

To understand why transient expression of DN-AtRop1 inhibits lesion expansion on potato leaves, the development of *P. infestans* mycelium around the inoculation sites was firstly detected using trypan blue staining. As shown in Figure 2C, compared to the sites of CA-AtRop1 expression, fewer mycelia were observed around the sites where DN-AtRop1 was transiently expressed (Figure 2C). Importantly, significant mycelium development was observed at the sites of CA-AtRop1 expression and the control sites, however, fewer mycelia was detected at DN-AtRop1 expression sites, indicating that mycelium development was inhibited to a certain extent by DN-AtRop1 overexpression. Meanwhile, real-time PCR was performed to evaluate the biomass of *P. infestans*. This showed that *P. infestans* mycelium development at the CA-AtRop1 and DN-AtRop1 transient expression sites was significantly lower than control sites, with DN-AtRop1 expression sites showing the lowest biomass levels (Figure 2D). In conclusion, the small lesion size at DN-AtRop1 transient expression sites could be correlated with limited growth of *P. infestans*.

**Figure 3:**
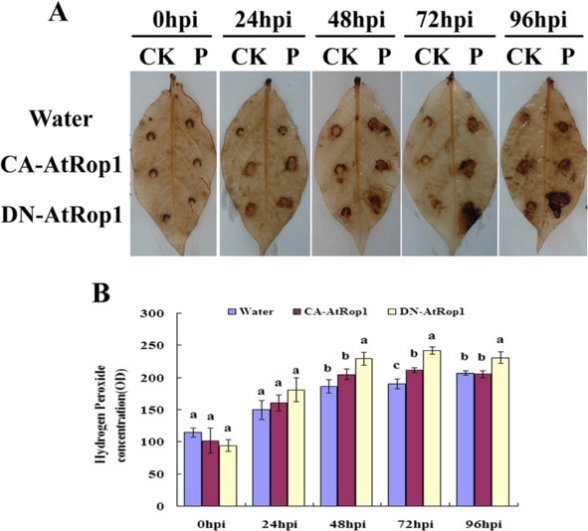
**The accumulation of H**_**2**_ **O**_**2**_ **at transient expression sites after inoculation with** ***P. infestan*** **. A**. The accumulation of H_2_O_2_ was visualized by staining leaf segments transiently expressing CA-AtRop1 and DN-AtRop1 with diaminobenzidine (DAB) over time after inoculation with *P. infestans*. The right side of each leaf was inoculated with *P. infestans*, and the left side used as the control. **B**. The accumulation of H_2_O_2_ was quantified using the Quantity One (Bio-Rad) tool. Each value represents the average (centimeters^2^ ± standard deviation) of three lesions. Levels not labeled with the same letter are significantly different at the *P < 0.05* level based on the ANOVA.

Next, we investigated the possible mechanisms underlying the inhibition of *P. infestans* development in potato after DN-AtRop1expression. Recent studies showed a tight link between ROPs and ROS accumulation [37]. This prompted us to analyze H_2_O_2_ accumulation by DAB staining at the *P. infestans* infection sites. At the DN-AtRop1 expression sites, H_2_O_2_ was observed 48 hpi, accumulated dramatically by 72 hpi and was still at high levels at 96 hpi (Figure 3A). However, this was not the case at the CA-AtRop1expression sites. In Figure 3B, quantification of DAB staining showed that both constructs led to increased H_2_O_2_ levels after inoculation with *P. infestans*. However, accumulation of H_2_O_2_ was much higher at the DN-AtRop1 expression sites than that at the CA-AtRop1 expression sites. We propose that the high level of H_2_O_2_ accumulation at DN-AtRop1 expression sites may be one of the causes of the increased resistance to *P. infestans*, illustrated by the inhibition of *P. infestans* development and the smaller lesion size on potato leaves.

### DN-AtRop1 enhances potato resistance to *P. infestans*

**Figure 4:**
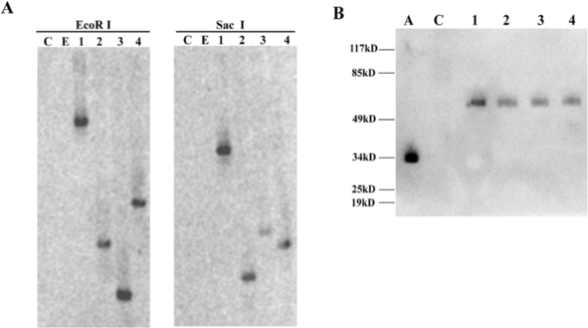
**The identification of positive potato transgenic lines. A**. Southern blot analysis of DN-AtRop1 transgenic lines with GFP probe. About 15 μg of the genomic DNA was digested with EcoR I and Sac I, electrophoresed on a 1.0% agarose gel, and blotted on to a positively charged nylon membrane. C: untransformed control plant, E: potato transformed with the empty vector p1300-221, 1–4: DN-AtRop1 potato transgenic lines (6, 7, 8, 11). **B**. Western blots analysis of the protein expression level of DN-AtROP1 in potato transgenic lines using an anti-GFP monoclonal antibody. A: positive control with only GFP protein, C: untransformed control plant. 1–4: DN-AtRop1 potato transgenic lines (6, 7, 8, 11).

To confirm the results observed in the transient expression system, we generated DN-AtRop1 transgenic potato lines *via A. tumefaciens* mediated transformation*.* The leaf disc method was used to generate transgenic lines, and positive transgenic lines were confirmed by Southern blot analysis (Figure 4A) and western blot using a commercial anti-GFP antibody (Figure 4B). DN-AtRop1 transgenic lines 6, 8 and 11 were chosen for further functional analysis.

**Figure 5:**
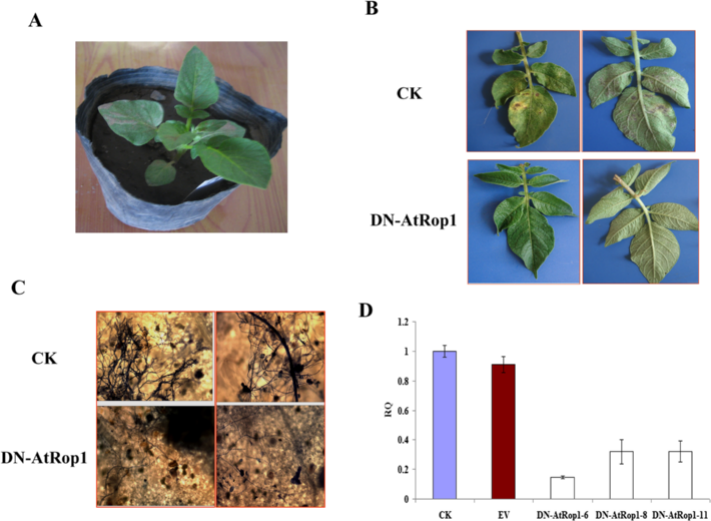
**Resistance of potato AtRop1 (DN-AtRop1) transgenic lines to** ***P. infestans*** **. A**. Four weeks old of transgenic (DN-AtRop1) potato. **B**. Symptoms on detached leaves of the DN-AtRop1 transgenic line after infection with *P. infestans*. Photographed at 96 hpi. **C**. Mycelial development on the leaves of DN-AtRop1transgenic lines (line 6 and line 11) after 96 hpi. The mycelium was stained with trypan blue. **D**. Relative quantification (RQ) by real-time PCR of lesion sites by comparing levels of the *P. infestans*-specific repetitive DNA sequence (as measure for fungal biomass) relative to the potato *EF-1α* gene (for equilibration) at 96hpi. Bars represent averages with standard deviation of three technical replicates. CK represents the wild-type potato and EV represents the potato transformed with the empty vector p1300-221.

We first checked symptom development on transgenic potato leaves after inoculation with *P. infestans*. As shown in Figure 5B, when the control line showed discoloration symptoms 96 hpi, lesions had not appeared on the DN-AtRop1 transgenic potato leaves. Mycelium development on transgenic potato leaves was detected by trypan blue staining. The sparse mycelium of *P. infestans* was detected in DN-AtRop1 transgenic potato leaves at 96 hpi, whereas, the control leaves expressed the empty vector, showed a mass of blue staining under the microscope (Figure 5C). *P. infestans* biomass in DN-AtRop1 transgenic potato leaves (lines 6, 8 and 11) was detected by real-time PCR. Quantitative analysis indicated reduced development of *P. infestans* in the three DN-AtRop1 transgenic lines compared to the control plants (Figure 5D), suggesting that the DN-AtRop1 transgene inhibits the development of *P. infestans* in potato leaves, thus increasing potato resistance to late blight.

### H_2_O_2_ is involved in potato AtRop1-mediated resistance to *P. infestans*

**Figure 6:**
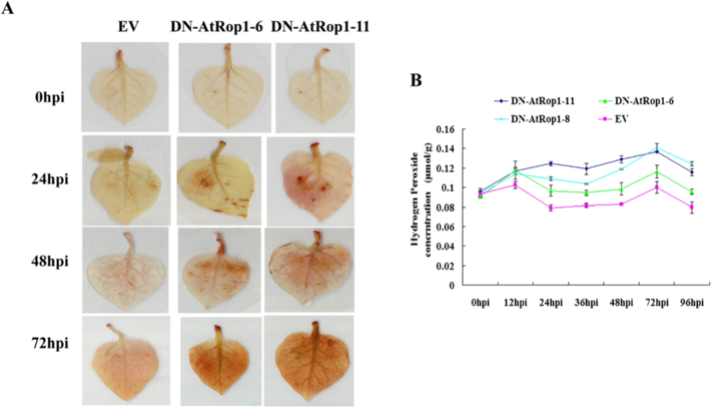
**H**_**2**_ **O**_**2**_ **accumulation in DN-AtRop1 transgenic lines after infection with** ***P. infestans*** **. A**. The accumulation of H_2_O_2_ was visualized by DAB staining on control and DN-AtRop1 transgenic plants at 0 hpi, 24 hpi, 48 hpi and 72 hpi. EV: transfomed with empty vector set up as control; DN-AtRop1-6 and DN-AtRop1-11 were two positive transgenic lines. **B**. The accumulation of H_2_O_2_ was quantified over time using a standard curve of titanylsulfate method. Each value is the mean of three replications of transgenic plants leaves of lines 6, 8 and 11.

In order to verify that H_2_O_2_ plays a key role in resistance of potato DN-AtRop1 transgenic lines to *P. infestans*, we analyzed H_2_O_2_ levels in the transgenic lines via DAB staining. Brown color was observed in DN-AtRop1 transgenic leaves at 24 hpi, whereas no signal was detected in control plants. Accumulation of H_2_O_2_ was observed in DN-AtRop1 transgenic plants at 48 hpi with higher levels at 72 hpi, while the control plants only showed a slight increased in brown color (Figure 6A). Quantification of H_2_O_2_ levels (based on the standard curve method of titanylsulfate) showed that the accumulation of H_2_O_2_ followed the same temporal pattern in the DN-AtRop1 and control lines, but levels were significantly higher in DN-AtRop1 transgenic lines (Figure 6B). Compared with control, all transgenic lines tested had higher H_2_O_2_ levels from 12hpi to 96hpi after inoculation suggesting that AtRop1 negatively regulated potato resistance via H_2_O_2_ accumulation.

### AtRop1 negatively regulats H_2_O_2_ accumulation via a potato NADPH oxidase homologues

**Figure 7:**
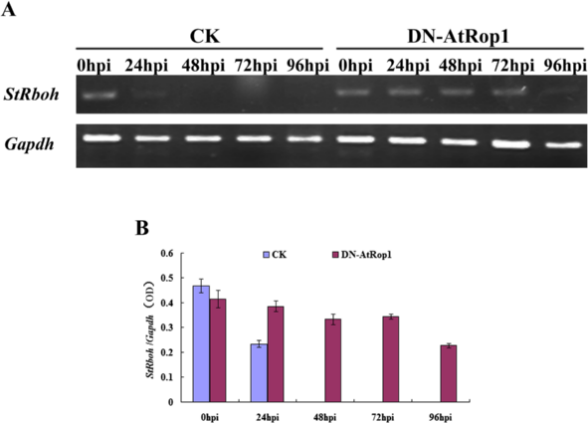
**Transcript expression of the** ***Strboh-D*** **gene in DN-AtRop1 transgenic plants after inoculation with** ***P. infestans*** **. A**. The transcript expression profile of the *Strboh-D* gene over time in DN-AtRop1 transgenic and control plants after *P. infestans* infection. Equal loading of cDNA was monitored by amplification of *Gapdh*. **B**. The relative expression level of the *Strboh-D* gene to *Gapdh* was quantified over time after inoculation with *P. infestans*.

We detected the accumulation of H_2_O_2_ after inoculation with *P. infestans* in DN-Rop1 transgenic lines. This data prompted us to check whether membrane-located Strboh (a NADPH oxidase homologue in potato) is the main regulator of H_2_O_2_ production in these transgenic lines. RT-PCR was performed using primers derived from *Strboh-D*, as shown in Figure 7A and B; transcript levels of Strboh-D were compared in DN-AtRop1 and control plants at different time points after inoculation (Figure 7B). In control plants, expression decreased by 24 hpi and was not detected at 48 hpi. However, in DN-AtRop1 transgenic plants, mRNA levels of *Strboh-D* gene remained constant at 24 hpi, decreasing slightly by 72 hpi, to almost half the control level by 96hpi (Figure 7A). These results suggest that the membrane localised *Strboh-D* gene may cause the accumulation of H_2_O_2_ in DN-AtRop1 transgenic lines after inoculation with *P. infestans*.

### JA may play a role in the resistance of DN-AtRop1 transgenic potato to *P. infestans*

**Figure 8:**
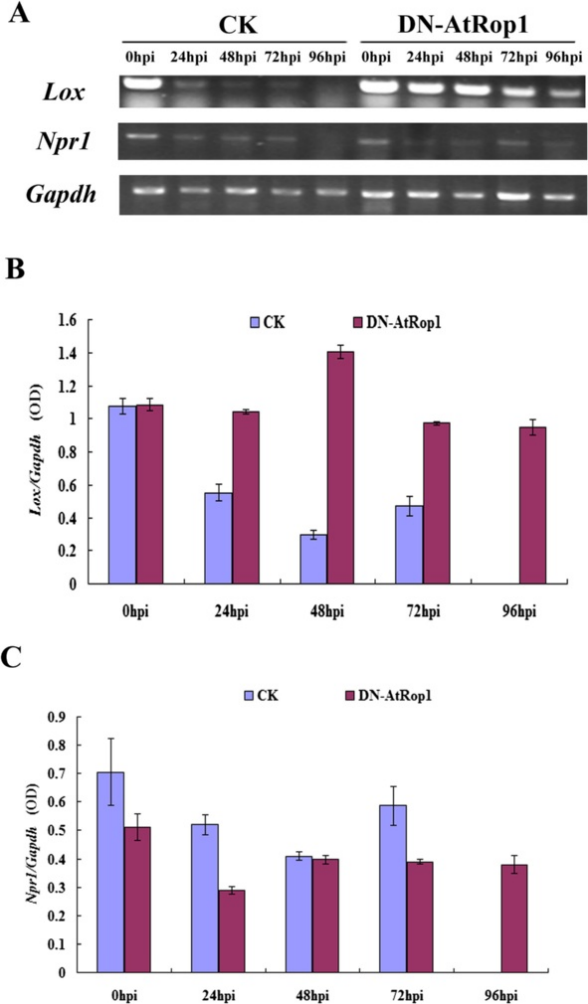
**Transcript expression profile of the** ***Lox*** **and** ***Npr1*** **genes in DN-AtRop1 transgenic plants after inoculation with** ***P. infestans*** **. A**. Transcript expression profile of the *Lox* and *Npr1* genes over time in DN-AtRop1 transgenic and control plants after *P. infestans* infection. Equal loading of cDNA was monitored by amplification of *Gapdh*. **B**. The relative expression level of the *Lox* gene to *Gapdh* was quantified over time. **C**. The relative expression level of the *Npr1* gene to *Gapdh* was quantified over time.

SA and JA are major signaling hormones involved in the establishment of plant resistance to the pathogens. To check whether JA- and SA-dependent signaling pathways were involved in DN-AtRop1-associated resistance to *P. infestans*, we analyzed the transcript levels of SA and JA marker genes. These marker genes were *Npr1*, a central component of the SA signaling pathway and up-regulated by SA accumulation, and *Lox*, which encodes a key enzyme in the JA biosynthetic pathway. As shown in Figure 8A and B, *Lox* transcript levels decreased dramatically in control plants by 24 hpi and were not detectable at 96 hpi, whereas in DN-AtRop1 transgenic plants, *Lox* mRNA remained constant until 48 hpi and decreased gradually afterwards. The transcript profile of *Npr1* was also studied. Quantification of expression data indicated that the *Npr1* basal level in DN-AtRop1 plants was slightly lower than in control plants (Figure 8C), and decreased further by 24hpi after inoculation with *P. infestans*. These results indicated that JA signaling may be involved in AtRop1-mediated potato resistance to late blight.

## Discussion

### Subcellular localization of the AtRop1 protein

Using a transient expression system, the subcellular localization of AtROP1 was investigated using GFP tagging in tobacco epidermal cells. Our results showed that mGFP:AtRop1 was mainly localized at the PM (plasma membrane) (Figure 1). In mammalian and plant cells, NADPH oxidase is responsible for the generation of H_2_O_2_ and is also located at the PM [61,62]. This raises the possibility that AtROP1 may regulate a NADPH oxidase homologue at the PM, thus facilitating the accumulation of H_2_O_2._ The same result was obtained in rice, where OsRac1 is also located at the PM and regulates H_2_O_2_ production, thus enhancing rice resistance to *M. grisea* [29-31]*.* The PM is proposed to be the platform for conveying signal transduction from small G proteins to downstream factors, thus enhancing the establishment of potato defense responses.

### AtRop1 negatively regulates potato resistance to late blight *via* accumulation of H_2_O_2_

Small GTPases of the RAC/ROP family play important roles in the establishment of plant defense responses [48,63]. In rice, overexpression of *OsRac1* enhanced host resistance by increasing H_2_O_2_ levels [29,51]; silencing this gene in rice led to the development of larger lesions than in control plants after infection with a rice blast pathogen [64]. Further studies on OsRac1function revealed that a constitutively active form of OsRac1(*CA-OsRac1*) promotes H_2_O_2_ production [29], whereas a dominant negative form of OsRac1 (*DN-OsRac*1) leads to the suppression of H_2_O_2_ [51]. Additionally, expressing dominant negative forms of human *Rac1* (DN-*hRac1)* in soybean or *OsRac1* in rice cell suspensions also leads to the suppression of H_2_O_2_ production in response to an elicitor treatment. This suggests the existence of a positive regulation mechanism by small G proteins on plant resistance mediated by H_2_O_2_ accumulation [39,51,65]. However, small G proteins may play more complex roles indicated by the expression of a dominant negative form of OsRac1 (*DN-OsRac1*) in tobacco. This led to induced H_2_O_2_ accumulation after infection with *Tobacco mosaic virus* (TMV) [66] and overexpression of *NtRac5* (Rac5 cloned from *Nicotiana tabacum*) in tobacco led to lower levels of H_2_O_2_ [67].We showed that the transient and stable expression of a dominant negative form of the Arabidopsis *AtRop1* (*DN-AtRop1*) gene in potato led to a decrease in the development of *P. infestans* at the infection sites. This suggests a negative regulation mechanism of AtRop1 on potato resistance to late blight. H_2_O_2_ was found to be a key player in the signaling pathway associated with AtRop1-mediated potato resistance. The overexpression of a negative form of AtRop1 resulted in the accumulation of H_2_O_2,_ and enhanced potato resistance to *P. infestans* infection. H_2_O_2_ is known to be a key player during the establishment of plant resistance [48,63]. The rapid production of H_2_O_2_, which triggers the HR in the infected areas, was observed in plants infected by non-host or avirulent strains of pathogens. In our study, overexpression of *DN-AtRop1* promoted the accumulation of H_2_O_2_ after *P. infestans* infection, which possibly triggered an HR in the infected area, thus limiting the development of *P. infestans*, and resulting in smaller lesions (Figure 2 and Figure 5). Therefore the increase in H_2_O_2_ accumulation may result in enhanced resistance of transgenic potato lines to late blight. Our data are in perfect agreement with what was observed in tobacco lines overexpressing *DN-OsRac1*, which also exhibited smaller lesions after TMV infection [66].

### H_2_O_2_ accumulation in DN-AtRop1 transgenic lines is mediated by NADPH oxidase

Generally, the production of H_2_O_2_ can be achieved either by mitochondrial electron transport during aerobic respiration or by oxidoreductases and metal-catalyzed oxidation. The activation of a membrane-associated NADPH oxidase constitutes the main source of H_2_O_2_ accumulation [68,69]. Strong induction of StRBOH which is a homologue of the NADPH oxidases catalytic subumit is observed not only in potato leaves, but also in potato tubers after infection with *P. infestans* [70]. In our study, sustained increased expression of *StRboh* in potato transgenic lines was observed after inoculation with *P. infestans*. However, its expression was suppressed dramatically in control plants. This indicates that the accumulation of H_2_O_2_ in the transgenic lines was likely to be caused by the sustained expression of the membrane-localisted *StRboh*. Meanwhile, the plasma membrane localization of both AtRop1 and StRboh suggests that the PM may be the platform connecting AtRop1 and *StRboh*, thus facilitating the accumulation of H_2_O_2_. Catalase is a major H_2_O_2_ scavenging enzyme inside plant cells, and hydrolyses H_2_O_2_ into H_2_O and O_2_ to protect plant cells from deleterious effects of H_2_O_2_ [71]. However, we did not observe differences in the transcript levels of catalase between control and transgenic lines after infection with *P. infestans*(data not shown), suggesting that H_2_O_2_ accumulation is mainly caused by potato homologues of PM-localized NADPH oxidases.

### JA is involved in potato resistance regulated by AtRop1

SA and JA are important signaling molecules in plant responses to pathogens. To check whether they were involved in the increased resistance of DN-AtRop1 transgenic lines to *P. infestans*, the transcript level of marker genes in these two pathways was estimated by RT-PCR. Our data showed that *Lox* gene expression was induced in DN-AtRop1 transgenic lines during *P. infestans* infection, indicating that the JA pathway was activated by DN-AtRop1 overexpression, whereas the SA signaling pathway was partially suppressed in this study.

Our data differ from previous reports showing that the SA signaling pathway was activated, and the JA signaling pathway was inhibited to a certain extent during *P. infestans* invasion of the resistant potato cultivar Zihuabai [72]. In addition, SA was found not only to regulate basal defense of potato against *P. infestans*, but was also involved in the establishment of systemic acquired resistance of potato induced by BABA [55,73]. However, Rosah and his colleague found that PAMP responses in potato required both SA and JA, indicating that JA is required for potato resistance establishment [6]. So, AtRop1 overexpression may activate PAMP-induced resistance pathways, thus enhancing potato resistance to *P. infestans*. A recent study established that JA and SA signaling pathways act antagonistically in potato [72]. The decreased *Npr1* transcript levels and the concomitant increase in JA after inoculation with *P. infestans* suggest a conservation of the antagonistic effects of SA and JA. In addition to SA and JA, there are other hormones involved in plant resistance, such as ethylene (ET), brassinosteroids, Cytokinins (CKs), and auxin. Among them, the brassinosteroids and CKs were shown to enhance the plant resistance to pathogens through NPR1 [74-76], a master regulator in the SA signaling pathway. Ethylene was confirmed to be antagonistic to SA signaling pathway [52], while auxin may lead to enhanced susceptibility to biotrophic pathogens by affect SA biosynthesis [77]. Our data have shown that expressing DN-AtRop1 led to decreased *Npr1* transcript levels after infection with *P. infestans*, indicating that AtRop1 may positively regulated the NPR1 expression in potato.

## Conclusion

We verified that AtRop1 could regulate potato resistance to late blight. The DN-AtRop1 appears to positively impact potato resistance to late blight, through activation of membrane-associated NADPH oxidase, thus facilitating H_2_O_2_ accumulation. We propose that H_2_O_2_ accumulation in turn limits *P. infestans* development, leads to smaller lesion size and enhances potato resistance to late blight. Our results also indicate that JA may be involved in AtRop1-mediated potato resistance to *P. infestans*.

## Methods

### Plasmid construction

The constructs pLG-CARop1, DNRop1 and Rop1, and the pBI121empty vector were kindly provided by Dr. Zhao Yan (Institute of Genetics and Developmental Biology, Chinese Academy of Sciences, Beijing). The constructs were digested with *EcoR*I and *Sac*I, then blunt-end and ligated with the empty vector pBI121 to construct three expression vectors: pBIG-CARop1, pBIG-DNRop1 and pBIGRop1. The PCR identification of *A. tumefaciens* containing pBIG-CARop1, pBIG-DNRop1 and pBIG-Rop1 were performed by using the GFP primers 5′-GAC GTA AAC GGC CAC AAG TT-3′ and 5′-GAA CTC CAG CAG GAC CAT GT-3′.

### Subcellular localization of AtRop1

*Agrobacterium tumefaciens* (strain LB4404) containing the expression vector (pBIG-CARop1/pBIG-DNRop1/ pBIG-Rop1) and the empty vector (GFP only) were grown for approximately 48 h in 5 ml LB medium contained 10 mM MES (pH 5.7), 20 μM acetosyringone and 100 mg/L rifampicin. Agrobacterium were collected and resuspended in 5% sucrose at a concentration of OD_600_ = 2.0, followed by infiltration into tobacco leaves using a syringe without the needle. After transient expression, the tobacco leaves were placed in the dark for 72 h. The GFP signal was detected in tobacco epidermal cells via confocal microscope (Carl Zeiss LSM-510).

### Preparation of zoospore suspensions of *P. infestans*

*Phytophthora infestans* mating type A1 was kindly provided by Prof. Zhang Ruofang (Potato Engineering Center of Inner Mongolia University). After culturing on sterilized oat grains at 18°C for 2–3 weeks, the sporangia of *P. infestans* were washed with sterile water and kept at 4°C for 4–6 hours to facilitate zoospore release. Zoospores were filtered through 1–2 layers of sterilized gauze, and the concentration determined using a haemocytometer under the microscope. The solution of zoospores was diluted to 5 × 10^5^ zoospore/mL for all inoculations [3].

The potato leaves were inoculated with 20 μL zoospore suspension (5 × 10^5^/mL) and incubated at 20°C with 85% humidity for resistance evaluation and determination of H_2_O_2_ accumulation [78]. Zoospore suspensions were also spread onto the abaxial surface of transgenic potato leaves with a sterile brush and kept at 20°C to detect H_2_O_2_ production and assays of mycelium biomass.

### *Agrobacterium tumefaciens* mediated transient expression

*Agrobacterium tumefaciens* (strain LB4404) carrying the AtRop1 mutants (CA-AtRop1 and DN-AtRop1) were grown for approximately 48 h in LB medium contained 10 mM MES (pH 5.7) and 20 μM acetosyringone. Following centrifugation, bacteria were resuspended in a infiltration medium that contained 10 mM MgCl_2_, 10 mM MES (pH 5.7) and 150 μM acetosyringone at a concentration of OD_600_ = 0.5 (optical density 600 nm). The bacteria were infiltrated into detached potato leaves using a syringe without needle. Twenty-four hours later, 20 Μl of the zoospore suspension(5 × 10^5^/mL)was inoculated at the infiltration sites. The resistance of potato to *P. infestans* was evaluated using lesion area of the inoculated leaves at different time points. Lesion area was estimated by counting the length × width of the lesion. Every experiment was repeated three times.

### Quantitative and qualitative determination of H_2_O_2_ production

The staining method 3, 3 ′-diaminobenzidine (DAB) was used to detect the accumulation of H_2_O_2_. The DAB solution was freshly prepared to avoid auto-oxidation. Leaf segments were floated in 0.1% (w/v) DAB solution (pH 3.8) and kept at 25°C in dark. After 8 h, the leaves were transferred into 95% ethanol and bleached by boiling for 30 min. Leaves were then stored in glycerol-ethanol (1/4 v/v) solution until photographed. The brown color due to DAB polymerization represented the accumulation level of H_2_O_2_ [79]. Color quantification data of DAB was evaluated by Quantity One (Bio-Rad, American) tools.

The titanium sulfate standard curve method was used to quantify the concentration of H_2_O_2_. The H_2_O_2_ and titanium sulfate generated yellow peroxidate-Ti compound precipitate. This problem was solved by treating with sulphuric acid; the color represents the concentration of H_2_O_2,_. Values were collected at OD_415_ nm and were linearly related to H_2_O_2_ concentration [80]. To generate the standard curve of H_2_O_2_, 30% standard H_2_O_2_(0, 0.1, 0.2, 0.4, 0.6, 0.8, 1.0 μM)was reacted with 5% titanium sulfate (w/v) in reaction buffer that contained ammonia and acetone, then solved with 2 M sulphuric acid. The regression equation was obtained through the formula: Y = 0.8144X + 0.025, R^2^ = 0.9953 by using values collected at OD_415_ nm through spectrophotometry(TU-1901)and used as standard curve. Then, 0.1 g of ground potato leaves were collected at 0, 24, 36, 48, 72 and 96 hpi, in liquid nitrogen and the powder transferred into 1.5 mL centrifuge tubes containing 1 mL prechilled acetone(4°C)to extract H_2_O_2_ . After centrifugation for 10 min at 6000 rpm, the 100 μL supernatant was used to react with 5% titanium sulfate (w/v). After the mixture was solved in 2 M sulphuric acid, the H_2_O_2_ concentration over time (0, 12, 24, 36, 48, 72 and 96 hpi) was detected using a UV Spectrophotometer at OD_415_ nm.

### Trypan blue staining to detect mycelium development

Trypan blue staining was used to detect the development of *P. infestans* mycelium. Infected potato leaf segments were transferred into 95% ethanol and boiled for 30 min to bleach the leaves. Leaves were then infiltrated with 0.5% (w/v) trypan blue solution for 4 hours at 25°C. After washing the samples with water for three times, the mycelium was visualized under the microscope [81].

### Quantify the development of *P. infestans* on potato leaves

Quantitative real time PCR was applied to determine the growth of *P. infestans* on potato plants using a lightcycler 480II(Roche, American) in combination with the qPCR kit SYBR Select Master Mix (ABI, American). DNA was extracted by using the hexa-decyl tri-methyl ammonium bromide (CTAB) method [82]. The primer PIO8-3-3F (5-CAA TTC GCC ACC TTC TTC GA-3) and PIO8-3-3R (5-GCC TTC CTG CCC TCA AGA AC-3) was used to amplify and detect a *P.infestans*-specific repetitive DNA sequence [83] and primer StEF-1-F(5-GTG TGT TAC GAG AAC TTG CTT TAC T-3) and StEF-1-R(GGA ACT ATG TAT TTT GCC ACC GTC CTG) was used to amplify potato *EF-1α* gene as an endogenous control.

### Plant materials and transformation

Potato plants *Solanum tuberosum cv* “Shepody” were grown on Murashige and Skoog (MS) medium *in vitro* at a photo flux density of 3000–4000 Lx, 60-85% relative humidity, and a 16 h/12 h day and night cycle at 25°C. A single colony of Agrobacteria LB4404 was cultured in a shaker culture of 10 mL of LB medium containing kanamycin (100 mg/L) and rifampin (100 mg/L) and kept at 28°C, 220 rpm for 48 h. The leaf veins of 3 ~ 4 weeks old potato seedlings leaves were cut, then floated in MS liquid medium that contained bacterial suspension (the bacterial concentration was OD_600_ = 0.5) and kept in the dark for 2–3 days. The leaves were transferred onto MS solid medium after drying on sterile filter paper. After 2 days, the leaves were transformed onto MS medium contained 0.5 mg/L indolylacetic acid (IAA), 2.0 mg/L 6-benzyl aminopurine (6-BA), 2.5 mg/L gibberellic acid (GA3), 25 mg/L hygromycin and 500 mg/L cefotaxime to induce adventitious buds. Four weeks later, regenerated potato shoots were grown on regeneration medium containing 25 mg/L hygromycin and 500 mg/L cefotaxime for selection. The transgenic plants were identified by Southern blot and Western blot.

### Southern blot analysis

Potato genomic DNA was extracted using the CTAB method [82]. For DNA extraction, 0.2 g of freshly harvested leaves was ground in liquid nitrogen. The powder was suspended in 1 mL of DNA extraction buffer (50 mM Tris–HCl pH 8.0, 10 mM EDTA, 700 mM NaCl, 1% CTAB solution, 0.5% PVP) and incubated at 65°C for 30 minutes. DNA was extracted by mixing with an equal volume of chloroform: isoamyl alcohol (24:1) and centrifuged at 12,000 rpm for 10 min at 4°C. The supernatant was removed to a fresh tube and precipitated overnight with equal volumes of ice-cold isopropanol and 0.1 volume 3 M sodium acetate in −20°C. The mixture was centrifuged at 12,000 rpm for 10 min at 4°C. The pellet was washed with 70% ethanol twice, then dried completely and dissolved in TE buffer. DNA was purified with RNAse and quantified by UV Spectrophotometry.

The GFP probe for Southern blotting was generated according to the instruction of DIG DNA Labeling and Detection Kit II (Roche, American) based on its sequence. After genomic DNA was digested with EcoR I and Sac I, the products were separated on 1.0% agarose gels, then transferred onto a nylon membrane. The membrane was washed with 2× SSC (0.3 M NaCl, 30 mM citrate sodium, pH 7.0), then twice with 0.1% SDS (5 min), followed by washing twice with 0.5 × SSC once and twice with 0.1% SDS (15 min) at 65°C. The membrane was baked at 80°C for 2 h to cross-link the DNA with the nylon membrane. The hybridization of the membrane with the labeled GFP probe was performed overnight in hybridization buffer (provided by DIG DNA Labeling and Detection Kit II) at 42°C. After hybridization, the membrane was washed with washing buffer (0.1 M maleic acid, 0.15 M NaCl, 0.3% Tween 20, pH 7.5) for 5 min; followed by incubation with blocking solution (provided by DIG DNA Labeling and Detection Kit II) for 30 min. The membrane was then incubated with Anti-Digoxigenin-AP (1:5000 dilutions) for 30 min. The membrane was washed with washing buffer twice, and equilibrated in detection buffer (0.1 M Tris–HCl, 0.1 M NaCl, pH 9.5) for 5 min. The membrane was stained with 1 ml CSPD, and incubated at 25°C for 5 min, then analysed ueing a Chemi XT4 (Syugene, American).

### Western-blot analysis

Transgenic potato leaves were ground in liquid nitrogen and the powder transferred into 1 mL protein isolation buffer (pH 6.0), which contained 5 mM K_3_PO_4_, 2.5% sucrose, 0.1% β-mercaptoethanol, and 0.5 mM phenylmethyl sulfonylfluoride. The crude extract was centrifuged at 4°C, 12,000 rpm for 20 min. The supernatant was collected and mixed with loading buffer. Proteins were separated by SDS-PAGE and transferred to a nitrocellulose membrane. The nitrocellulose membrane was blocked with 5% non-fat dry milk in PBST(Phosphate Buffered Saline Tween-20) buffer and incubated with an anti-GFP monoclonal antibody (1:5000 dilution, Sangon, China) and HRP-conjugated anti-rat secondary antibody (1:3000 dilution, Sangon, China) to detect the signal by Chemi XT4 (Syugene, American).

### RT-PCR

Total RNA was extracted from potato transgenic leaves using the RNA iso Reagent (TaKaRa, Japan). The quality of RNA was examined on a 1.2% agarose gel. The cDNA for RT-PCR was generated using AMV transcriptase (TaKaRa, Japan) according to the manufacturer’s instructions. The specific primers used for RT-PCR analysis were as follows:

- StRboh-D-F(5-AGC TGC AGA ATA CGC AGC GTT GA-3)
- StRboh-D-R(5-GGC ATT GAA ACC GGT GAG CTT GT-3)
- Npr1-F(5-TGC TGC CAT GCG TAA CGA ACC A-3)
- Npr1-R(5-TGG ACC AAA ACT TGG CCC CAC A-3)
- Lox-F(5-AGA ACT TTG CTC TTC TTG CAA G-3)
- Lox-R(5-GGT AAT ATT CAT TGT GTC CCG-3)
- GAPDH-F (5-TGG ACA ATG GAA GCA CCA TGA GC-3)
- GAPDH-R (5-TGC TTG ACC TGC TGT CAC CAA GA-3).

## References

[1] Zhao ZZ, Sun X (2009). Research status and control strategy of potato late blight. China Plant Prot.

[2] Sun ZK, Niu C, Yang SS (2006). Research of potato late blight. Life Sci Res.

[3] Wang BL (2005). Induced Expression Profiling of Potato Genes Associated with Quantitative Resistance to Late Blight and Preliminary Exploring of Quantitative Resistance Mechanism.

[4] Fry WE, Goodwin SB (1997). Resurgence of the Irish potato famine fungus. Bioscienece.

[5] Vleeshouwers VG, Van Dooijeweert W, Govers F, Kamoun S, Colon LT (2000). The hypersensitive response is associated with host and nonhost resistance to Phytophthora infestans. Planta.

[6] Halim VA, Altmann S, Ellinger D, Eschen-Lippold L, Miersch O, Scheel D, Rosahl S (2009). PAMP-induced defense responses in potato require both salicylic acid and jasmonic acid. Plant J.

[7] Halim VA, Hunger A, Macioszek V, Landgraf P, Nürnberger T, Scheel D, Rosahl S (2004). The oligopeptide elicitor Pep-13 induces salicylic acid-dependent and -independent defense reactions in potato. Physiol Mol Plant Pathol.

[8] Yamamizo C, Kuchimura K, Kobayashi A, Katou S, Kawakita K, Jones JDG, Noriyuki D, Hirofumi Y (2006). Rewiring mitogen-activated protein kinase cascade by positive feedback confers potato blight resistance. Plant Physiol.

[9] Shibata Y, Kawakita K, Takemoto D (2010). Age-related resistance of Nicotiana benthamiana against hemibiotrophic pathogen Phytophthora infestans requires both ethylene and salicylic acid-mediated signaling pathways. Mol Plant Microbe Interact.

[10] Shi X, Tian Z, Liu J, van der Vossen EA, Xie C (2012). A potato pathogenesis-related protein gene, StPRp27, contributes to race-nonspecific resistance against Phytophthora infestans. Mol Biol Rep.

[11] Cohen Y, Gisi U, Niderman T (1993). Local and systemic protection against Phytop hthor a infestans induced in potato and tomato plants by jasmonic acid and jasmonic methyl ester. Phytopathology.

[12] Parada LF, Tabin CJ, Shih C, Weinberg RA (1982). Human EJ bladder carcinoma oncogene is homologue of Harvey carcoma virus Ras gene. Nature.

[13] Hall A (1998). Rho GTPases and the actin cytoskeleton. Science.

[14] Chant J, Stowers L (1995). GTPase cascades choreographing cellular behavior: movement, morphogenesis and more. Cell.

[15] Takai Y, Sasaki T, Matozaki T (2001). Small GTP-binding proteins. Physiol Rev.

[16] Settleman J (2001). Rac’n Rho: the music that shapes a developing embryo. Dev Cell.

[17] Hall A, Nobes CD (2000). Rho GTPases: molecular switches that control the organization and dynamics of the actin cytoskeleton. Philos Trans R Soc Lond B Biol Sci.

[18] Chen X, Friml J (2014). Rho-GTPase-regulated vesicle trafficking in plant cell polarity. Biochem Soc Trans.

[19] Zheng ZL, Yang ZB (2000). The Rop GTPase: an emerging signaling switch in plants. Plant Mol Biol.

[20] Yang ZB (2002). Small GTPase: versatile signaling switches in plants. Plant Cell.

[21] Vernoud V, Horton AC, Yang ZB, Nielsen E (2003). Analysis of the small GTPase gene superfamily of Arabidopsis. Plant Physiol.

[22] Gu Y, Wang Z, Yang Z (2004). ROP/RAC GTPase: an old new master regulator for plant signaling. Curr Opin Plant Biol.

[23] Xu J, Scheres B (2005). Cell polarity: ROPing the ends together. Curr Opin Plant Biol.

[24] Craddock C, Lavagi I, Yang Z (2012). New insights into Rho signaling from plant ROP/Rac GTPases. Trends Cell Biol.

[25] Bischoff F, Molendijk A, Rajendrakumar CS, Palme K (1999). GTP-binding proteins in plants. Cell Mol Life Sci.

[26] Winge P, Brembu T, Kristensen R, Bones AM (2000). Genetic structure and evolution of RAC-GTPases in Arabidopsis thaliana. Genetics.

[27] Christensen TM, Vejlupkova Z, Sharma YK, Arthur KM, Spatafora JW, Albright CA, Meeley RB, Duvick JP, Quatrano RS, Fowler JE (2003). Conserved subgroups and developmental regulation in the monocot rop gene family. Plant Physiol.

[28] Humphries JA, Vejlupkova Z, Luo A, Meeley RB, Sylvester AW, Fowler JE, Smith LG (2011). ROP GTPases act with the receptor-like protein PAN1 to polarize asymmetric cell division in maize. Plant Cell.

[29] Kawasaki T, Henmi K, Ono E (1999). The small GTP-binding protein rac is a regulator of cell death in plants. Proc Natl Acad Sci U S A.

[30] Kawasaki T, Koita H, Nakatsubo T, Hasegawa K, Wakabayashi K, Takahashi H, Umemura K, Umezawa T, Shimamoto K (2006). Cinnamoyl-CoA reductase, a key enzyme in lignin biosynthesis, is an effector of small GTPase Rac in defense signaling in rice. Proc Natl Acad Sci U S A.

[31] Ono E, Wong HL, Kawasaki T, Hasegawa M, Kodama O, Shimamoto K (2001). Essential role of the small GTPase Rac in disease resistance of rice. Proc Natl Acad Sci U S A.

[32] Oda T, Hashimoto H, Kuwabara N, Akashi S, Hayashi K, Kojima C, Wong HL, Kawasaki T, Shimamoto K, Sato M, Shimizu T (2010). Structure of the N-terminal regulatory domain of a plant NADPH oxidase and its functional implications. J Biol Chem.

[33] Schultheiss H, Hensel G, Imani J, Broeders S, Sonnewald U, Kogel KH, Kumlehn J, Hückelhoven R (2005). Ectopic expression of constitutively activated RACB in barley enhances susceptibility to powdery mildew and abiotic stress. Plant Physiol.

[34] Schultheiss H, Preuss J, Pircher T, Eichmann R, Hückelhoven R (2008). Barley RIC171 interacts with RACB in planta and supports entry of the powdery mildew fungus. Cell Microbiol.

[35] Jung YH, Agrawal GK, Rakwal R, Kim JA, Lee MO, Choi PG, Kim YJ, Kim MJ, Shibato J, Kim SH, Iwahashi H, Jwa NS (2006). Functional characterization of OsRacB GTPase-a potentially negative regulator of basal disease resistance in rice. Plant Physiol Biochem.

[36] Chen L, Shiotani K, Togashi T (2010). Analysis of the Rac/Rop small GTPase family in rice: expression, subcellular localization and role in disease resistance. Plant Cell Physiol.

[37] Hassanain HH, Sharma Y, Moldovan L, Khramtsov V, Berliner LJ, Duvick JP, Goldschmidt-Clermont PJ (2000). Plant rac proteins induce superoxide production in mammalian cells. Biochem Biophys Res Commun.

[38] Potikha TS, Collins CC, Johnson DI, Delmer DP, Levine A (1999). The involvement of hydrogen peroxide in the differentiation of secondary walls in cotton fibers. Plant Physiol.

[39] Park J, Choi HJ, Lee S, Lee T, Yang Z, Lee Y (2000). Rac-related GTP-binding protein in elicitor-induced reactive oxygen generation by suspension-cultured soybean cells. Plant Physiol.

[40] Wang AR, Chen X, Zhang DM, Chen HH, Lu GD, Wang ZH (2008). Effects of different Arabidopsis ROPs on multiplication of Pseudomonas syringae pv. tomato DC3000. J Fujian Agric For Univ (Natural Science Edition).

[41] Schultheiss H, Dechert C, Kogel KH, Hückelhoven R (2002). A small GTP-binding host protein is required for entry of powdery mildew fungus into epidermal cells of barley. Plant Physiol.

[42] Pathuri IP, Zellerhoff N, Schaffrath U, Hensel G, Kumlehn J, Kogel KH, Eichmann R, Hückelhoven R (2008). Constitutively activated barley ROPs modulate epidermal cell size, defense reactions and interactions with fungal leaf pathogens. Plant Cell Rep.

[43] Hoefle C, Huesmann C, Schultheiss H (2011). A barley ROP GTPase ACTIVATING PROTEIN associates with microtubules and regulates entry of the barley powdery mildew fungus into leaf epidermal cells. Plant Cell.

[44] Schiene K, Puhler A, Niehaus K (2000). Transgenic tobacco plants that express an antisense construct derived from a Medicago sativa cDNA encoding a Rac-related small GTP-binding protein fail to develop necrotic lesions upon elicitor infiltration. Mol Gen Genet.

[45] Leonard MK, Hannah FB, Christine S, Wimmer D, Korte J, Schmitz U, Niehaus K, Colditz F (2012). Silencing of the Rac1 GTPase MtROP9 in medicago truncatula stimulates early mycorrhizal and oomycete root colonizations but negatively affects rhizobial infection. Plant Physiol.

[46] Doke N (1983). Involvement of superoxide anion generation in the hypersensitive response of potato tuber tissues to infection with an incompatible race of Phytophthora infestans and to the hyphal wall components. Physiol Plant Pathol.

[47] Yoshioka H, Numata N, Nakajima S, Katou S, Kawakita K, Rowland O, Jones JD, Doke N (2003). Nicotiana benthamiana gp91phox homologs NbrbohA and NbrbohB participate in H2O2 accumulation and resistance to Phytophthora infestans. Plant Cell.

[48] Lamb C, Dixon RA (1997). The oxidative burst in plant disease resistance. Annu Rev Plant Physiol Plant Mol Biol.

[49] Delledonne M, Zeier J, Marocco A, Lamb C (2001). Signal interactions between nitric oxide and reactive oxygen intermediates in the plant hypersensitive disease resistance response. Proc Natl Acad Sci U S A.

[50] Kim DS, Jeun Y, Hwang BK (2014). The pepper patatin-like phospholipase CaPLP1 functions in plant cell death and defense signaling. Plant Mol Biol.

[51] Ono E, Wong HL, Kawasaki T, Hasegawa M, Kodama O, Shimamoto K (2001). Essential role of the small GTPase Rac in disease resistance of rice. Proc Natl Acad Sci U S A.

[52] Glazebrook J (2005). Contrasting mechanisms of defense against biotrophic and necrotrophic pathogens. Annu Rev Phytopathol.

[53] Kunkel BN, Brooks DM (2002). Cross talk between signaling pathways in pathogen defense. Curr Opin Plant Biol.

[54] Durrant WE, Dong X (2004). Systemic acquired resistance. Annu Rev Phytopathol.

[55] Halim V, Eschen-Lippold L, Altmann S, Birschwilks M, Scheel D, Rosahl S (2007). Salicylic acid is important for basal defense of Solanum tuberosum against Phytophthora infestans. Mol Plant-Microbe Interact.

[56] Coquoz JL, Buchala AJ, Meuwly P, Métraux JP (1995). Arachidonic acid induces local but not systemic synthesis of salicylic acid and confers systemic resistance in potato plants to Phytophthora infestans and Alternaria solani. Biochem Cell Biol.

[57] Penninckx IA, Thomma BP, Buchala A, Métraux JP, Broekaert WF (1998). Concomitant activation of jamonate and ethylene response pathways is required for induction of a plant defensin gene in Arabidopsis. Plant Cell.

[58] Spoel SH, Koornnef A, Claessens SM, Korzelius JP, Van Pelt JA, Mueller MJ, Buchala AJ, Métraux JP, Brown R, Kazan K, Van Loon LC, Dong X, Pieterse CM (2003). NPR1 modulates cross-talk between salicylate- and jasmonatedependent defense pathways through a novel function in the cytosol. Plant Cell.

[59] Truman W (2007). Arabiodopsis systemic immunity uses conserved defence signaling pathways and is mediated by jasmonates. Proc Natl Acad Sci U S A.

[60] Van Wees SCM, Chang H-S, Zhu T, Glazebrook J (2003). Characterization of the early response of Arabidopsis to Alternaria brassicicola infection using expression profiling. Plant Physiol.

[61] Segal AW, Abo A (1993). The biochemical basis of the NADPH oxidase of phagocytes. Trends Biochem Sci.

[62] Keller T, Damude HG, Werner D, Doerner P, Dixon RA, Lamb C (1998). A plant homolog of the neutrophil NADPH oxidase gp91phox subunit gene encodes a plasma membrane protein with Ca2+ binding motifs. Plant Cell.

[63] Neill S, Desikan R, Hancock J (2002). Hydrogen peroxide signaling. Curr Opin Plant Biol.

[64] Letian C, Kenji S, Takashi T, Miki D, Aoyama M, Wong HL, Kawasaki T, Shimamoto K (2010). Analysis of the Rac/Rop small GTPase family in rice: expression, subcellular localization and role in disease resistance. Plant Cell Physiol.

[65] Suharsono U, Fujisawa Y, Kawasaki T, Iwasaki Y, Satoh H, Shimamoto K (2002). The heterotrimeric G protein α subunit acts upstream of the small GTPase Rac in disease resistance of rice. Proc Natl Acad Sci U S A.

[66] Wolfgang M, Keiko Y, Daniel FK (2005). Involvement of the small GTPase Rac in the defense responses of tobacco to pathogen. MPMI.

[67] Morel J, Fromentin J, Blein JP, Simon-Plas F, Elmayan T (2004). Rac regulation of NtrbohD, the oxidase responsible for the oxidative burst in elicited tobacco cell. Plant J.

[68] Lambeth JD (2000). Regulation of the phagocyte respiratory burst oxidase by protein interactions. J Biochem Mol Biol.

[69] Bokoch GM, Diebold BA (2002). Current molecular models for NADPH oxidase regulation by Rac GTPase. Blood.

[70] Gao L, Tu ZJ, Millett BP, Bradeen JM (2013). Insights into organ-specific pathogen defense responses in plants: RNA-seq analysis of potato tuber-Phytophthora infestans interactions. BMC Genomics.

[71] Frank VB, Eva V, James FD, Dirk I (2001). The role of active oxygen species in plant signal transduction. Plant Sci.

[72] Na R, Zhang Z, Yu X, Zhang X, Yang F, Zhao J (2013). ROS and salicylic acid (SA) play roles on the resistance establishment of the potato cultivar Zihuabai to Phytophthora infestans. J Plant Dis Prot.

[73] Eschen-Lippold L, Altmann S, Rosahl S (2010). DL-beta-aminobutyric acid-induced resistance of potato against Phytophthora infestans requires salicylic acid but not oxylipins. Mol Plant Microbe Interact.

[74] Dong X (2004). NPR1, all things considered. Curr Opin Plant Biol.

[75] Divi UK, Rahman T, Krishna P (2010). Brassinosteroid-mediated stress tolerance in Arabidopsis shows interactions with abscisic acid, ethylene and salicylic acid pathways. BMC Plant Biol.

[76] Choi J, Huh SU, Kojima M, Sakakibara H, Paek KH, Hwang I (2010). The cytokinin-activated transcription factor ARR2 promotes plant immunity via TGA3/NPR1-dependent salicylic acid signaling in Arabidopsis. Dev Cell.

[77] Navarro L, Bari R, Achard P, Lisón P, Nemri A, Harberd NP, Jones JD (2008). DELLAs control plant immune responses by modulating the balance of jasmonic acid and salicylic acid signaling. Curr Biol.

[78] Hu C (2003). Screening of Potato Clones with Horizontal Resistance to Late Blight (Phytophthora Infestans).

[79] Hernández JA, Ferrer MA, Jiménez A, Barceló AR, Sevilla F (2001). Antioxidant systems and O2.-/H2O2 production in the apoplast of pea leaves. Its relation with salt-induced necrotic lesions in minor veins. Plant Physiol.

[80] Su GX, An ZF, Liu YL, Liu Y (2005). Light promotes the synthesis of lignin through the production of H2O2 mediated by diamine oxidases in soybean hypocotyls. J Plant Phys.

[81] Wang Z, Mao H, Dong C, Ji R, Cai L, Fu H, Liu S (2009). Overexpression of Brassica napus MPK4 enhance resistance to sclerotinia sclerotiorum in oilseed rape. MPMI.

[82] Doyle JJ, Doyle JL (1987). A rapid DNA isolation procedure for small quantities of leaf tissue. Phytochem Bull.

[83] Lennart EL, Grit R, Michael S, Göbel C, Feussner I, Rosahl S (2007). Reduction of divinyl ether-containing polyunsaturated fatty acids in transgenic potato plants. Phytochemistry.

